# Prognostic significance of vascular endothelial growth factor expression in human ovarian carcinoma

**DOI:** 10.1054/bjoc.2000.1228

**Published:** 2000-06-15

**Authors:** G H Shen, M Ghazizadeh, O Kawanami, H Shimizu, E Jin, T Araki, Y Sugisaki

**Affiliations:** Department of Molecular Pathology, Institute of Gerontology, Nippon Medical School, 1–396 Kosugi-cho, Nakahara-ku, Kawasaki, 211, Japan; Department of Obstetrics and Gynecology, Nippon Medical School, Tokyo, Japan; Division of Surgical Pathology, Nippon Medical School, Tokyo, Japan

**Keywords:** ovarian tumours, vascular endothelial growth factor, microvessel density, angiogenesis, disease stage, prognosis

## Abstract

The influence of vascular endothelial growth factor (VEGF) expression and microvessel density (MVD) on prognosis and the relationship between VEGF expression and MVD in ovarian carcinoma are not well defined. We studied VEGF expression in parallel with MVD by immunohistochemistry in 94 ovarian tumours (64 malignant, 13 borderline, and 17 benign) and correlated the results with the clinicopathologic prognostic factors of the disease to clarify their significance in this disease. Assessment of VEGF mRNA isoforms by RT-PCR was also performed. Of the malignant, borderline, and benign ovarian tumours respectively, two (3%), four (31%) and 16 (94%) were negative, 31 (48%), seven (54%) and one (6%) had low expressions, and 31 (48%), two (15%) and none (0%) had high expressions of VEGF. There were significant associations between the VEGF expression and disease stage (*P* = 0.002), histologic grade (*P* = 0.0004), and patient outcome (*P* = 0.0002). MVD did not correlate significantly with the clinicopathologic parameters. Likewise, no correlation was found between MVD and VEGF expression. The survival of patients with high VEGF expression was significantly worse than that of patients with low and negative VEGF expression (*P* = 0.0004). Multivariate analysis revealed that disease stage and VEGF expression were significant and independent prognostic indicators of overall survival time (*P* = 0.008 and *P* = 0.006 respectively). These findings suggest that in conjunction with the established clinicopathologic prognostic parameters of ovarian carcinoma, VEGF expression may enhance the predictability of patients at high risk for tumour progression who are potential candidates for further aggressive therapy. © 2000 Cancer Research Campaign


*British Journal of Cancer* (2000) **83**(2), 196–203
© 2000 Cancer Research Campaign
doi: 10.1054/ bjoc.2000.1228, available online at http://www.idealibrary.com on IDEAL®

### Prognostic significance of vascular endothelial growth factor expression in human ovarian carcinoma

**GH Shen[1,2], M Ghazizadeh[1], O Kawanami[1], H Shimizu[1], E Jin[1], T Araki[2] and Y Sugisaki[3]**

[1]Department of Molecular Pathology, Institute of Gerontology, Nippon Medical School, 1–396 Kosugi-cho, Nakahara-ku, Kawasaki, Japan 211; [2]Department of Obstetrics and Gynecology, Nippon Medical School, Tokyo, Japan; [3]Division of Surgical Pathology, Nippon Medical School, Tokyo, Japan

**Summary** The influence of vascular endothelial growth factor (VEGF) expression and microvessel density (MVD) on prognosis and the relationship between VEGF expression and MVD in ovarian carcinoma are not well defined. We studied VEGF expression in parallel with MVD by immunohistochemistry in 94 ovarian tumours (64 malignant, 13 borderline, and 17 benign) and correlated the results with the clinicopathologic prognostic factors of the disease to clarify their significance in this disease. Assessment of VEGF mRNA isoforms by RT-PCR was also performed. Of the malignant, borderline, and benign ovarian tumours respectively, two (3%), four (31%) and 16 (94%) were negative, 31 (48%), seven (54%) and one (6%) had low expressions, and 31 (48%), two (15%) and none (0%) had high expressions of VEGF. There were significant associations between the VEGF expression and disease stage ($P = 0.002$), histologic grade ($P = 0.0004$), and patient outcome ($P = 0.0002$). MVD did not correlate significantly with the clinicopathologic parameters. Likewise, no correlation was found between MVD and VEGF expression. The survival of patients with high VEGF expression was significantly worse than that of patients with low and negative VEGF expression ($P = 0.0004$). Multivariate analysis revealed that disease stage and VEGF expression were significant and independent prognostic indicators of overall survival time ($P = 0.008$ and $P = 0.006$ respectively). These findings suggest that in conjunction with the established clinicopathologic prognostic parameters of ovarian carcinoma, VEGF expression may enhance the predictability of patients at high risk for tumour progression who are potential candidates for further aggressive therapy. © 2000 Cancer Research Campaign

**Keywords**: ovarian tumours; vascular endothelial growth factor; microvessel density; angiogenesis; disease stage; prognosis

Ovarian carcinoma has the highest mortality rate among gynaecological malignancies. About two-thirds of patients have already advanced disease at the time of diagnosis. Established prognostic indicators of ovarian carcinoma currently include disease stage, histologic grade, residual tumour volume, and response to chemotherapy. The identification of prognostic factors that, in association with established clinicopathologic parameters, predict which patients are at high risk for the development of tumour progression, is significantly helpful in better treatment and improvement of the survival of patients with ovarian carcinoma. Angiogenesis is an essential requirement for tumour growth and metastasis and basically depends on the production of angiogenic factors by host and/or tumour cells (Folkman, 1986; 1990). It has been shown that in animal models, without angiogenesis, tumours grow as in situ and will not expand beyond 2–3 mm in diameter (Folkman, 1986). The importance of angiogenesis in tumour progression has been highlighted by studies showing that the angiogenic potential of tumours assessed by tumour microvessel density (MVD) directly correlates with poor prognosis (Weidner et al, 1991; Weidner and Folkman, 1996).

Various growth factors have been shown to stimulate angiogenesis in physiological and pathological conditions, including neoplastic disease. Among these, vascular endothelial growth factor (VEGF) has been shown to play a major role in the proliferation and migration of endothelial cells, providing nourishment to the growing tumours and allowing the tumour cells to establish continuity with the host vasculature (Ferrara, 1995). VEGF is a 34–45 kDa heparin-binding glycoprotein which was originally identified as a hyperpermeability factor and subsequently found to be a potent mitogen for the endothelial cells (Ferrara, 1995; Senger et al, 1983; Ferrara and Hanzel, 1989; Keck et al, 1989). It induces neovascularization through its specific receptors flk-1 (KDR) and flt-1 (Terman et al, 1992; deVries et al, 1992). By alternating splicing of mRNA, four major isoforms of VEGF with 121, 165, 189, and 206 amino acids may be identified (Tischer et al, 1991). These isoforms have different bioavailabilities due to their different heparin-binding activity. VEGF 121 does not bind heparin and is secreted as a freely soluble protein. VEGF 165 is a basic, heparin-binding protein, abundant in many human tissues and tumours, and is also secreted, but to a lesser degree than the 121 isoform. The longer isoforms with 189 and 206 amino acids have a greater affinity to heparin and are incorporated in the extracellular matrix. Inhibition of VEGF activity by neutralizing antibodies, or by the introduction of dominant negative VEGF receptors into endothelial cells of tumour-associated blood vessels, resulted in the inhibition of tumour growth, and tumour regression, indicating that VEGF is a principal initiator of tumour angiogenesis (Kim et al, 1993; Millaure et al, 1994).

Recent studies have demonstrated a significant correlation between VEGF expression and MVD in malignant tumours arising from several organs (Ferrara, 1999). In ovarian carcinomas, VEGF and its receptors flt-1 and flk-1 (KDR) have been detected at mRNA and/or protein levels (Olson et al, 1994; Boocock et al, 1995; Abu-Jawdeh et al, 1996; Paley et al, 1997; Sowter et al,

*Received 28 October 1999*
*Revised 14 February 2000*
*Accepted 28 February 2000*

*Correspondence to*: M Ghazizadeh

1997; Yamamoto et al, 1997; Fujimoto et al, 1998). It was found that patients with early stage ovarian carcinoma and increased VEGF expression had poorer prognosis (Paley et al, 1997). Moreover, strong VEGF expression was suggested to play an important role in the tumour progression of ovarian carcinoma (Yamamoto et al, 1997). In these studies, no attempt has been made to assess the relationship between VEGF expression and MVD. Studies addressing such an interrelationship are very limited (Nakanishi et al, 1997; Hartenbach et al, 1997; Orre and Rogers, 1999). In one study, a significant association was found between the MVD and VEGF expression (Nakanishi et al, 1997). In contrast, two other studies found no correlation between MVD and VEGF expression (Hartenbach et al, 1997; Orre and Rogers, 1999). Furthermore, studies using multivariate statistical analyses to assess the potential prognostic value of MVD and VEGF expression in ovarian carcinoma are rare. In a study using multivariate analysis in early-stage ovarian carcinomas, VEGF overexpression was found to be the strongest independent variable predictive of a poor survival (Paley et al, 1997). Another study showed that only disease stage was significant prognostic factor and VEGF expression was not an independent prognostic indicator (Yamamoto et al, 1997). VEGF expression was also found to be correlated significantly with disease-free survival in patients with invasive serious ovarian carcinomas (Garzetti et al, 1999*a*; 1999b). Thus, the influence of VEGF expression and MVD on prognosis and the relationship between VEGF expression and MVD in ovarian carcinoma are still controversial.

The purpose of this study was to clarify the significance of VEGF expression and MVD in relation to established clinicopathologic prognostic factors of ovarian carcinoma.

### MATERIALS AND METHODS

This study is composed of 94 diagnosed patients with epithelial ovarian tumours who underwent surgery. The representative tissue specimens from the lesions included 64 consecutively operated ovarian carcinomas, 13 borderline and 17 benign ovarian tumours (Table 1). The ages of patients ranged from 21–88 years (median, 54 years). Serial 4-μm sections from the tissues were prepared. A section from each tissue was stained with haematoxylin and eosin to verify the tissue and confirm the diagnosis. Histological types and grades were determined using the World Health Organization Criteria (Serov et al, 1973), and the stage of tumours was assessed according to the International Federation of Gynecology and Obstetrics staging system (Peterson, 1988). All carcinoma patients on this study received postoperative chemotherapy with various regimens for a high-risk early stage (stage I, grade 3; stage IC; any stage II) or advanced diseases (stages III and IV). The clinicopathologic characteristics of patients are shown in Table 2. Tumour sizes ranged from 5–30 cm in greater diameter (median, 12 cm). Of the 64 ovarian carcinoma patients, 43 were alive with no evidence of the disease, six were alive with disease, and 15 were dead of the disease at the time of analysis. The median follow-up for all patients ($n$ = 64) was 31 months (range, 3–120), and for surviving patients ($n$ = 49) was 40.5 months (range, 16–120).

### Immunohistochemistry

Immunostaining for VEGF and factor VIII-related antigen (specific for endothelial cells) was performed using the avidin-biotin-peroxidase complex method. Endogenous peroxidase was blocked in deparaffinized tissue sections by 3% hydrogen peroxide in methanol for 20 min. After washing with phosphate buffer saline (PBS), pH 7.4, tissue-nonspecific binding sites were blocked by 10% normal goat serum for 20 min. The sections were then incubated with affinity-purified rabbit polyclonal anti-human VEGF (Santa Cruz Biotechnology Inc, Santa Cruz, CA, USA) at 1:100 dilution or anti-human factor VIII-related antigen (Dako Co., Carpinteria, CA, USA) at 1:200 dilution overnight at 4°C. Anti-human VEGF antibody had been raised against an amino-terminal peptide (amino acids 1–20) of VEGF molecule which recognizes all splice variants of VEGF. This antibody has been widely used and accepted for immunohistochemical localization of VEGF (Boocock et al, 1995; Yamamoto et al, 1997; Garzetti et al, 1999*a*; 1999*b*; Orre and Rogers 1999). After washes with PBS, biotinylated anti-rabbit IgG (Vector Laboratories, Burlingame, CA, USA) was applied followed by washing and detection using the avidin-biotin-peroxidase complex (Dako Co). Diamino-benzidine was used as chromogen. The sections were counterstained briefly with Mayer's haematoxylin, dehydrated, and mounted. Negative controls included the use of a nonspecific rabbit immunoglobulin as the primary antibody. As positive control, we used sections from an adenocarcinoma of the lung previously found to be positive for VEGF expression and factor VIII-related antigen.

**Table 1** VEGF expression and microvessel density in ovarian tumours

| | | VEGF expression | | Microvessel density | |
|---|---|---|---|---|---|
| Variable | $n$ | Low + Neg | High | Low | High |
| Adenocarcinoma[a] | 64 | 31 + 2 | 31 | 26 | 38 |
| Serous | 29 | 14 + 0 | 15 | 9 | 20 |
| Mucinous | 12 | 8 + 0 | 4 | 4 | 8 |
| Endometroid | 5 | 1 + 1 | 3 | 2 | 3 |
| Clear cell | 18 | 8 + 1 | 9 | 11 | 7 |
| Borderline tumour | 13 | 7 + 4 | 2 | 4 | 9 |
| Serous | 2 | 1 + 0 | 1 | 0 | 2 |
| Mucinous | 11 | 6 + 4 | 1 | 4 | 7 |
| Benign cystadenoma | 17 | 1 + 16 | 0 | 6 | 11 |
| Serous | 5 | 0 + 5 | 0 | 2 | 3 |
| Mucinous | 9 | 1 + 8 | 0 | 2 | 7 |
| Endometroid | 3 | 0 + 3 | 0 | 2 | 1 |

The chi-square test and Fisher's exact test were used for comparison. [a]VEGF expression in adenocarcinoma vs borderline and benign tumours: $P$ = 0.02 and $P$ = 0.0002 respectively.

### Evaluation of immunostaining and microvessel counting

Evaluation of immunostained sections was done without knowledge of the clinical status of the patients. VEGF immunostainings were semiquantitated according to a method used by us previously (Ghazizadeh et al, 1997). After screening the whole section and selection of microscopic fields using a random point grid, the mean percentage of specific brown immunostained tumour cells for five microscopic fields at × 200 magnification (× 20 objective lens and × 10 ocular lens; 0.74 mm$^2$ per field) was determined and was assigned an arbitrary numerical score as: 0% = 0; 1–25% = 1; 26–50% = 2; 51–75% = 3; and 76–100% = 4. In addition, the mean intensity of immunostained areas based on the arbitrary numerical

© 2000 Cancer Research Campaign

**Table 2** Clinicopathologic characteristics, VEGF expression and microvessel density in ovarian carcinomas

| Variable | *n* | VEGF expression Low + Neg | VEGF expression High | *P*[a] | Microvessel density Low | Microvessel density High | *P*[a] |
|---|---|---|---|---|---|---|---|
| Age | | | | | | | |
| ≤ 60 yrs | 42 | 23 + 2 | 17 | 0.07 | 18 | 24 | 0.61 |
| > 60 yrs | 22 | 8 + 0 | 14 | | 8 | 14 | |
| Tumour size | | | | | | | |
| ≤ 10 cm | 20 | 11 + 0 | 9 | 0.71 | 8 | 12 | 0.94 |
| > 10 cm | 44 | 20 + 2 | 22 | | 18 | 26 | |
| Histologic type | | | | | | | |
| Serous | 29 | 14 + 0 | 15 | 0.68 | 9 | 20 | 0.21 |
| Mucinous | 12 | 8 + 0 | 4 | | 4 | 8 | |
| Endometroid | 5 | 1 + 1 | 3 | | 2 | 3 | |
| Clear cell | 18 | 8 + 1 | 9 | | 11 | 7 | |
| Histologic grade | | | | | | | |
| Low (1–2) | 46 | 28 + 2 | 16 | 0.0004 | 21 | 25 | 0.19 |
| High (3) | 18 | 3 + 0 | 15 | | 5 | 13 | |
| Disease stage | | | | | | | |
| Low (I–II) | 37 | 23 + 2 | 12 | 0.002 | 17 | 20 | 0.31 |
| High (III–IV) | 27 | 8 + 0 | 19 | | 9 | 18 | |
| Patient outcome | | | | | | | |
| Alive with no evidence of disease | 43 | 27 + 2 | 14 | 0.0002 | 18 | 25 | 0.77 |
| Died or alive with disease | 21 | 4 + 0 | 17 | | 8 | 13 | |

[a]The chi-square test and Fisher's exact test were used for comparison scores of none = 0, weak = 1, moderate = 2, and strong = 3 was recorded. The two values obtained for each section were then multiplied by each other to generate the semiquantitative value of VEGF antigen per tissue (range, 0–12).

For microvessel counting, each stained slide for factor VIII-related antigen was examined under low magnification (× 100) to identify the regions of highest vascular density (vascular 'hot spots') within the tumour. Microvessels were counted in five microscopic fields of highest vascular density at × 200 magnification (as above). The mean microvessel count was then calculated for each specimen, averaged between two independent observers, and was recorded as 'MVD'. Small vessels counted were capillaries, arterioles and venules, regardless of the presence or absence of lumens (Weidner et al, 1991). Larger arteries with thick smooth muscle walls and distended venous sinuses were excluded.

### Reverse transcription polymerase chain reaction (RT-PCR) analysis

Total RNA from ovarian tissues (one normal ovary, one serious cystadenoma, two serous papillary, one poorly differentiated and one mucinous adenocarcinoma of the ovary) was extracted using the acid guanidinium thiocyanate-phenol-chloroform extraction method. Complementary DNA was synthesized from 2 μg of total RNA primed with oligo$(dT)_{16}$ using Superscript II reverse transcriptase (Gibco BRL Ltd, Rockville, MD, USA) at 37°C for 1.5 h. Nested PCR was carried out using 1 μl of the cDNA and the following primers (5′–3′) that span the variable splice region of VEGF mRNA: (a) GCTACTGCCATCCAATCGAGACC (exon 3, forward); (b) GTT TCTGGATTAAGGACTGTTCTGTCG (exon 8, reverse); and (c) AATCCAATTCCAAGAGGGACCGTGC (exon 8, reverse). First-round amplification was carried out using 0.4 μmol $l^{-1}$ each of primers (a) and (c) for 15 cycles (1 min at 94°C, 2 min at 62°C, and 3 min at 72°C). Then 1 μl of the first round PCR products was used for second-round amplification with the nested primers (a) and (b). Amplification was for 30 cycles under the same conditions as for the first-round amplification. The PCR product was resolved by gel eletrophoresis and visualized by ethidium bromide staining. A glyceraldehyde 3-phosphate dehydrogenase (GAPDH) was used as the internal control. Using the described primers, the expected PCR product for each VEGF variant was calculated as: 440 bp, 572 bp, 644 bp, and 695 bp for isoforms of 121, 165, 189, and 206 respectively.

### Statistical analysis

VEGF expression and MVD in relation to various clinicopathologic factors were assessed using the chi-square test and Fisher's exact test. Correlation between VEGF expression and MVD was examined by Spearman rank correlation test. Cumulative survival probabilities of the patients were calculated by the Kaplan–Meier method and log-rank test. The results were analysed for the endpoint of overall survival. Survival times of patients still alive were censored with the last follow-up date. Cox proportional hazards regression analysis was used to identify parameters that had significant independent relation with overall survival time. All statistical analyses were done by the SPSS statistical software system. A probability (*P*) value of less than 0.05 was considered significant.

### RESULTS

### VEGF expression and microvessel density

VEGF immunoreactivity was observed mainly in the cytoplasm of tumour cells, and also frequently in stromal cells and macrophages (Figure 1). In benign mucinous cystadenomas, epithelial cells were mostly negative for VEGF staining, but the luteinized theca-like

 © 2000 Cancer Research Campaign

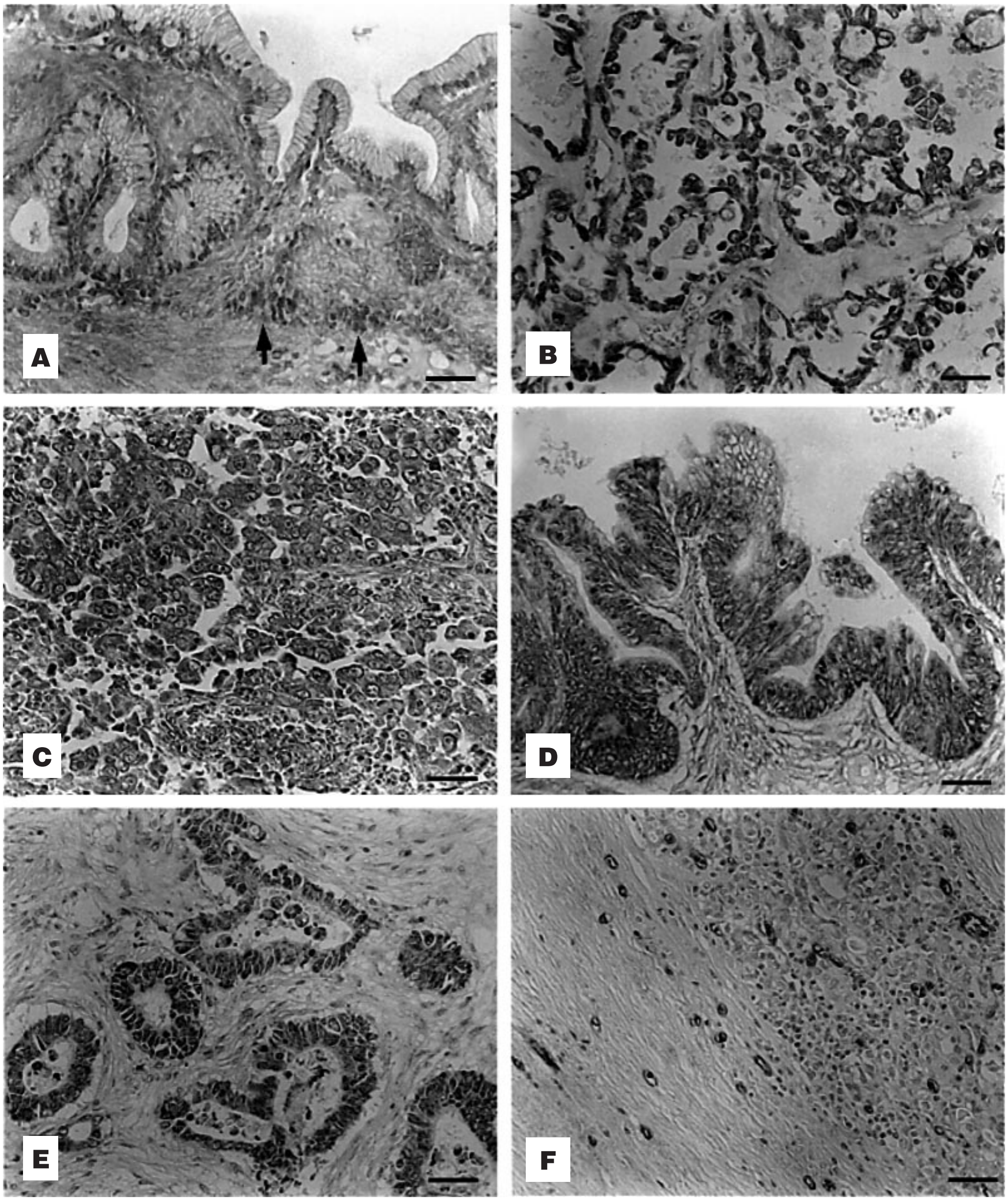

**Figure 1** Immunohistochemical staining for VEGF (**A** to **E**) and Factor VIII-related antigen (**F**) in ovarian tumours. (**A**) Benign mucinous cystadenoma showing lack of the staining in epithelial cells and presence of weak staining in luteinized theca-like cells in the subepithelial stromal region (arrows). (**B**) Serous papillary, (**C**) poorly differentiated serous and (**D**) well-differentiated mucinous cystadenocarcinomas, and (**E**) endometroid type adenocarcinoma, all showing moderate to strong cytoplasmic staining in the epithelial tumours cells. (**F**) Distinct visualization of intratumoural microvessels. Scale bars: 100 μm.

cells in the subepithelial stroma showed positive staining (Figure 1A). Negative control sections lacked immunostaining for VEGF, while positive control sections showed moderate to strong immunostainings for it. The median semiquantitative VEGF expression value for ovarian carcinomas was 3 (range: 0–8) which was designated as a cut-off limit for determining a low and negative (values 0–2) and high (values 3–8) expression. Of the 64 ovarian carcinomas, two (3%) were negative and 31 (48%) each had low and high VEGF expressions (Figure 1 B–E). Of the 13 borderline ovarian tumours, four (31%) were negative, seven (54%) had low and two (15%) had high VEGF expression. Of the 17 benign cystadenomas, 16 (94%) were negative, and one (6%) had low VEGF expression (Table 1).

Factor VIII-related antigen staining was observed in the endothelial cells lining intratumoural microvessels, however with variable intensities (Figure 1F). The mean MVD in benign, borderline, and malignant ovarian tumours were 38.88 ± 17.81, 37.54 ± 11.65, and 37.64 ± 16.08, respectively. The median MVD value for ovarian carcinomas was 32 (range: 9–85), which was designated as a cut-off limit for dividing the cases into two groups of low (< 32) and high (≥ 32) MVD for the statistical comparisons.

### RT-PCR ANALYSIS

RT-PCR was used to assess the differential expression of VEGF mRNA splice isoforms in normal ovary and a number of benign

*© 2000 Cancer Research Campaign*

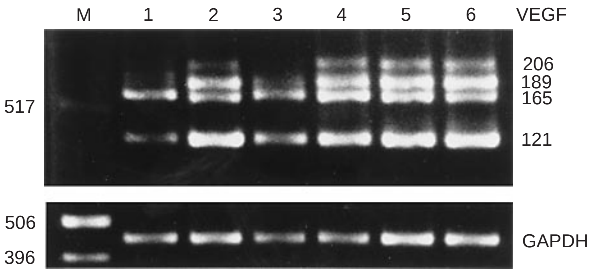

**Figure 2** VEGF mRNA expression in ovarian tissues showed four bands of 440, 572, 644, and 695 bps corresponding to VEGF isoforms of 121, 165, 189 and 206 respectively. M: molecular weight marker; Lane 1: normal ovary; Lane 2: serous cystadenoma; Lanes 3 and 5: serous papillary adenocarcinoma; Lane 4: poorly differentiated adenocarcinoma; Lane 6: mucinous adenocarcinoma. Note that all tissues expressed VEGF mRNA corresponding to the isoforms 121, 165, and 189.

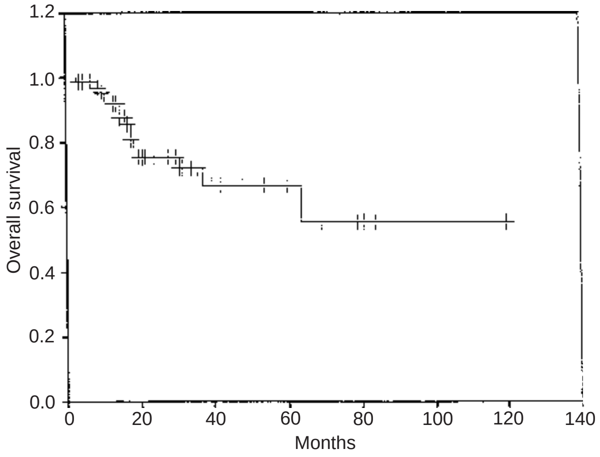

**Figure 3** The overall survival curve of ovarian carcinoma patients showing a 5-year survival time of 66.4%.

and malignant ovarian tumours from which frozen tissue samples were available. PCR amplification of cDNA prepared from frozen tissues of one normal ovary, one serous cystadenoma, two serous papillary, one mucinous, and one poorly differentiated adenocarcinoma of the ovary showed the expression of four bands of 440, 572, 644, and 695 bps corresponding to VEGF isoforms of 121, 165, 189 and 206 respectively (Figure 2). In normal ovary, the predominant isoforms were VPF/VEGF 165 and 121 respectively, with the lack of isoform 206. In cystadenoma and carcinoma of the ovary, all four isoforms were detected, however with an occasional lack of the longest isoform 206 in case of carcinoma (Figure 2, case No. 3). The predominant isoforms were, however, VEGF 121, 165, and 189 in that order.

### Clinicopathologic correlations of VEGF expression and microvessel density

A high VEGF expression was significantly associated with ovarian carcinomas as compared to benign ($P$ = 0.0002) or borderline ($P$ = 0.02) ovarian tumours (Table 1). No significant difference was found between the low and negative vs high VEGF expressions and patient age, tumour size, and histologic types (Table 2). However, histologic grade, disease stage, and patient outcome showed significant differences ($P$ = 0.0004, $P$ = 0.002, and

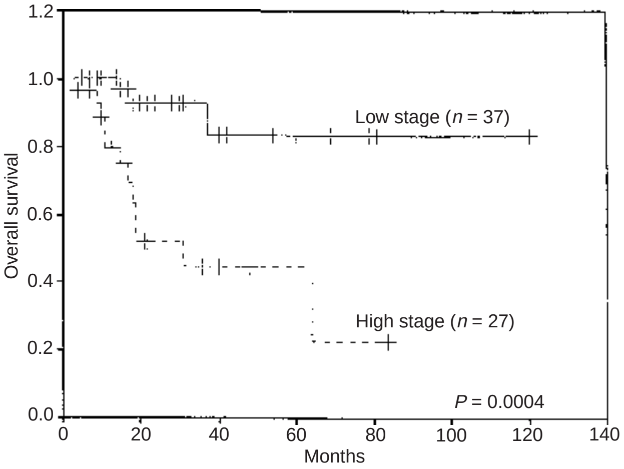

**Figure 4** Survival curves of ovarian carcinoma patients grouped according to low (I–II) and high (III–IV) disease stages showing a significant difference ($P$ = 0.0004).

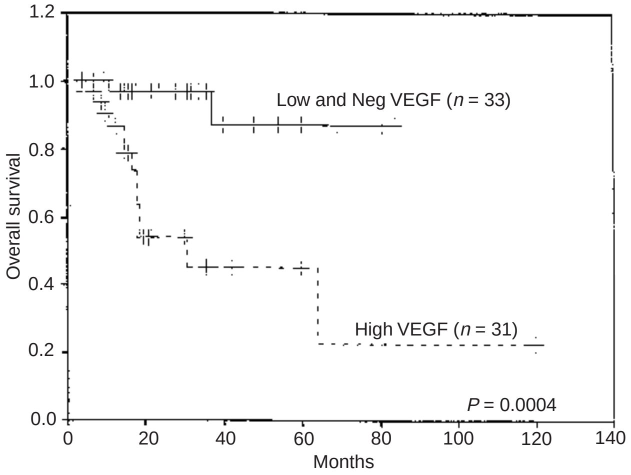

**Figure 5** Survival curves of ovarian carcinoma patients grouped according to low and negative (scores 0–2) versus high (scores > 3) VEGF expression showing a significant difference ($P$ = 0.0004).

$P$ = 0.0002 respectively). The mean MVD, as well as a high or low MVD, did not differ significantly among benign, borderline, and malignant ovarian tumours (Table 1). Likewise, no significant correlation was found between MVD and the clinicopathologic parameters in ovarian carcinomas (Table 2). Moreover, there was no correlation between VEGF expression and MVD (Spearman rank correlation coefficients: $r$ = 0.0173, $P$ = 0.892). We also examined whether any correlation exists between the two parameters within each set of histologic type of ovarian tumours and failed to find a significant correlation (data not shown).

To investigate whether VEGF expression and MVD predict the prognosis of patients with ovarian carcinomas, cumulative survival curves for the 64 patients with ovarian carcinomas were constructed according to the Kaplan–Meier method, and differences in survival were assessed with the log-rank test. The overall 5-year survival probability of the patients in our series was 66.4% (Figure 3). The survival rate of patients with a high disease stage (survivors, $n$ = 15) or a high VEGF expression in the tumour (survivors, $n$ = 18) was significantly worse than that of patients

*© 2000 Cancer Research Campaign*

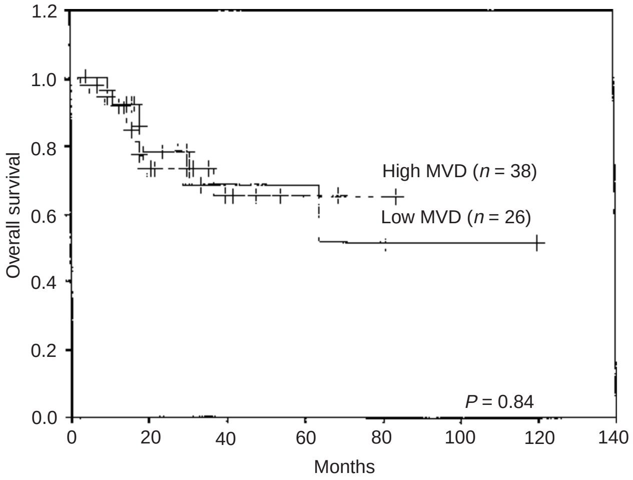

**Figure 6** Survival curves of ovarian carcinoma patients grouped according to low (<32) and high (≥32) microvessel density (MVD) showing no significant difference (*P* = 0.84).

with a low disease stage (survivors, *n* = 34) or a low or negative VEGF expression (survivors, *n* = 31; *P* = 0.0004; Figure 4 and 5). Further survival analysis of low-stage patients (*n* = 37) in relation to VEGF expression did not show a significant difference, because of the small number of events occurring in this subgroup. A high or low MVD did not correlate with the survival time of patients (Figure 6).

In a multivariate Cox proportional hazards regression analysis, VEGF expression and disease stage were found to be significant independent prognostic indicators of overall survival time (*P* = 0.006 and *P* = 0.008, Table 3).

**Table 3** Multivariate Cox proportional hazards regression analysis

| Variable | Wald chi-square | *P* |
|---|---|---|
| Microvessel density (low vs high) | 0.215 | 0.643 |
| Histologic grade (1–2 vs 3) | 2.261 | 0.132 |
| Disease stage (I–II vs III–IV) | 7.032 | 0.008 |
| VEGF (low and negative vs high) | 7.335 | 0.006 |

### DISCUSSION

The current retrospective study established that both VEGF expression and disease stage were independent significant prognostic factors for predicting the survival of patients with ovarian carcinomas. Overall immunoreactivity for VEGF was observed in 6% of benign cystadenomas, 69% of borderline tumours, and 96% of carcinomas. The frequency of high VEGF expression was significantly higher in carcinomas (48%) than that in benign (0%) or borderline (15%) tumours (*P* = 0.0002 and *P* = 0.02 respectively). Among the clinicopathologic parameters of ovarian carcinoma, the degree of VEGF expression also correlated significantly with the histologic grade, disease stage, and patient outcome. Particularly, patients with high VEGF expression had poorer survival times.

VEGF was first recognized as a vascular permeability factor that induced tumour ascites and was active in increasing blood vessel permeability, endothelial cell growth, and angiogenesis (Senger et al, 1983; Leung et al, 1989). It was also described as having a direct mitogenic action specific to vascular endothelial cells (Keck et al, 1989). In ovarian carcinomas, the expression of VEGF has been documented (Boocock et al, 1995; Abu-Jawdeh et al, 1996). An increased VEGF mRNA or protein expression has been correlated with a worse prognosis in patients with early and advanced stage ovarian carcinomas (Paley et al, 1997; Hartenbach et al, 1997). VEGF level has been shown to be elevated in cyst fluid of ovarian malignancy (Hazelton et al, 1999) and in ascitis fluid of a variety of malignancies (Kraft et al, 1999). Serum VEGF level has also been reported to be elevated in ovarian carcinoma patients (Kraft et al, 1999; Tempfer et al, 1998) and it was shown to be an independent prognostic factor (Tempfer et al, 1998). As our study was retrospective in nature, the serum VEGF values of the patients had not been measured to allow correlation with the immunohistochemical findings. In our study, immunostaining for VEGF was largely restricted to the carcinoma cells, which is consistent with the previous reports (Boocock et al, 1995; Abu-Jawdeh et al, 1996) and support the observed increased serum VEGF levels in ovarian cancer as well (Kraft et al, 1999; Tempfer et al, 1998). We also observed VEGF immunostaining in the luteinized theca-like cells in the subepithelial stroma, particularly in mucinous cystadenomas. This finding is in accord with the previous observation of strong VEGF expression in the luteinized cells of developing follicles and corpora lutea (Kamat et al, 1995), and accounts for a portion of VEGF production by the ovary in health and disease. We did not observe a significant correlation between VEGF expression and MVD in ovarian tumours. This finding is consistent with the previous observations from a study on a limited number of epithelial ovarian carcinomas showing that a high VEGF expression was associated with a poor overall survival, but no association was found between the VEGF expression and tumour MVD (Hartenbach et al, 1997). In one study, a significant association was found between MVD and VEGF expression in that the MVD of VEGF-rich tumours was significantly higher than that of VEGF-poor tumours (Nakanishi et al, 1997). In contrast, another study of serous and mucinous ovarian tumours suggested that VEGF may play a role in the control of angiogenesis in serous and benign tumours, but it does not seem to contribute to the higher MVD in mucinous tumours (Orre and Ragers, 1999). Therefore, we also sought to determine whether any correlation exists between VEGF expression and MVD within each set of histologic type of ovarian tumours, but failed to identify a significant correlation. The lack of correlation between VEGF expression and mean MVD observed in this study could be explained by differences in methodologies used for the assessment of VEGF and MVD. These include the use of serial tissue sections and the equal size of the examined tumour area for determining the expression level of VEGF and mean MVD employed in this study. These factors were not taken into account in many other studies.

In our series, the mean MVD values in benign, borderline, and malignant ovarian tumours were almost similar, and a high or low MVD did not correlate with other clinicopathologic parameters of ovarian carcinomas, including the survival time of patients (Table 2 and Figure 7). The prognostic independent influence of neovascularization has been reported in several types of human solid cancers (Weidner et al, 1996). In ovarian carcinomas, it has been demonstrated that analysis of neovascularization in advanced stage ovarian cancer may be a useful prognostic factor (Hollingsworth et al, 1995). However, recent results contradicted

© 2000 Cancer Research Campaign the putative association between increased MVD and poor prognosis in ovarian tumours (Orre et al, 1998). The result of our study is in accord with this notion. These findings suggest that in ovarian tumours, increased VEGF expression may not indicate an enhanced angiogenesis, but it may reflect growth activity of the tumour cells. In this context, a recent study by Garzetti et al (1999*b*) has shown a correlation between VEGF expression and MIB1 index, which is an important proliferative marker.

Several different isoforms of VEGF have been described that result from alternative splicing of its mRNA. To assess whether differential regulation of VEGF isoforms by ovarian carcinoma could account for its increased secretion, RT-PCR analysis was employed to find out which isoforms were expressed specifically in normal, benign, and malignant ovarian tissues. We found VEGF 121, 165, and 189 to be uniformly expressed in all ovarian tissues with the predominant isoforms being 165 and 121; and that the isoform 206 was occasionally lacking. The relative abundance of the different isoforms did not alter appreciably among different histologic types, or among normal, benign, and malignant ovarian tissues. Although many cases need to be examined before drawing any conclusion, our results on the small number of tissues examined is at least consistent with the results of VEGF mRNA isoform expression in a previous study of ovarian carcinoma cell lines (Boocock et al, 1995) and a recent study of normal ovary and ovarian carcinomas of various histologic types which showed the isoforms 165 and 121 to be predominant regardless of the histologic type and disease stage (Fujimoto et al, 1998).

In conclusion, our study signifies the prognostic value of VEGF expression in ovarian carcinoma, and suggests that in conjunction with the established clinicopathologic prognostic parameters, VEGF expression may help to more accurately predict patients at high risk for tumour progression who are potential candidates for aggressive therapy. The significance of high VEGF expression may be more applicable in patients with low-stage disease, since low-stage patients with high VEGF expression may benefit from additional therapy. This speculation awaits further studies and approval. Finally, prospective studies are warranted to establish the role of VEGF expression as a prognostic marker in ovarian carcinoma.

### ACKNOWLEDGEMENT

This work was accomplished during the period of a research fellowship grant from Sasagawa Foundation, Tokyo, Japan, to Gui Hua Shen MD, Presently at the Department of Obstetrics and Gynecology, Beijing Hospital, Beijing, Republic of China.

### REFERENCES

Abu-Jawdeh GM, Faix JD, Niloff J, Tognazzi K, Manseau E and Dvorak HF et al (1996) Strong expression of vascular permeability factor (vascular endothelial growth factor) and its receptor in ovarian borderline and malignant neoplasms. *Lab Invest* **74**: 1105–1115

Boocock CA, Charnock-Jones DS, Sharkey AM, McLaren J, Barker PJ and Wright KA et al (1995) Expression of vascular endothelial growth factor and its receptor fit and KDR in ovarian carcinoma. *J Natl Cancer Inst* **87**: 506–516

de Vries C, Escobedo JA, Ueno H, Houck K, Ferrara N and Williams LT (1992) The fms-like tyrosine kinase, a receptor for vascular endothelial growth factor. *Science* **255**: 989–991

Ferrara N and Hanzel WJ (1989) Pituitary folicular cells secrete a novel heparin-binding growth factor specific for vascular endothelial cells. *Biochem Biophys Res Commun* **161**: 851–858

Ferrara N (1995) The role of vascular endothelial growth factor in pathological angiogenesis. *Breast Cancer Res Treat* **36**: 127–137

Ferrara N (1999) Vascular endothelial growth factor: molecular and biological aspects. *Curr Top Microbiol Immunol* **237**: 1–30

Folkman J (1986) How is blood vessel growth regulated in normal and neoplastic tissue? G.H.A. Clowes Memorial Award Lecture. *Cancer Res* **46**: 467–473

Folkman J (1990) What is the evidence that tumours are angiogenesis-dependent? *J Natl Cancer Inst* **82**: 4–6

Fujimoto J, Sakaguchi H, Hirose R, Ichigo S and Tamaya T (1998) Biologic implications of the expression of vascular endothelial growth factor subtypes in ovarian carcinoma. *Cancer* **83**: 2528–2533

Garzetti GG, Ciavattini A, Lucarini G, Pugnaloni A, De Nictolis M and Amati S et al (1999*a*) Expression of vascular endothelial growth factor related to 72-kilodalton metalloproteinase immunostaining in patients with serous ovarian tumours. *Cancer* **85**: 2219–2225

Garzetti GG, Ciavattini A, Lucarini G, Pugnaloni A, De Nictolis M and Amati S et al. (1999*b*) Vascular endothelial growth factor expression as a prognostic index in serous ovarian cystadenocarcinomas: relationship with MIB1 immunostaining. *Gynecol Oncol* **73**: 396–401

Ghazizadeh M, Ogawa H, Sasaki Y, Araki T and Aihara K (1997) Mucin carbohydrate antigens (T, Tn, and sialyl-Tn) in human ovarian carcinoma: Relationship with histopathology and prognosis. *Hum Pathol* **28**: 960–966

Hartenbach EM, Olson TA, Goswitz JJ, Mohanraj D, Twiggs LB, Carson LF and Ramakrishnan S (1997) Vascular endothelial growth factor (VEGF) expression and survival in human epithelial ovarian carcinomas. *Cancer Lett* **121**: 169–175

Hazelton D, Nicosia RF and Nicosia SV (1999) Vascular endothelial growth factor levels in ovarian cyst fluid correlate with malignancy. *Clin Cancer Res* **5**: 823–829

Hollingsworth HC, Kohn EC, Steinberg SM, Rothenberg ML and Merino MJ (1995) Tumour angiogenesis in advanced stage ovarian carcinoma. *Am J Pathol* **147**: 33–41

Kamat BR, Brown LF, Manseau EJ, Senger DR and Dvorak HF (1995) Expression of vascular permeability factor/vascular endothelial growth factor by human granulosa and theca lutein. *Am J Pathol* **146**: 157–165

Keck PJ, Hauser SD, Krivi G, Sanzo K, Warren T and Feder J, et al (1989) Vascular permeability factor: an endothelial cell mitogen related to PDGF. *Science* **246**: 1309–1312

Kim KJ, Li B, Winer J, Armanini M, Gillet N, Phillips HS and Ferrara N (1993) Inhibition of vascular endothelial growth factor induced angiogenesis suppresses tumor growth in vivo. *Nature* **362**: 841–844

Kraft A, Weindel K, Ochs A, Marth C, Zmija J, Schumacher P et al. (1999) Vascular endothelial growth factor in sera and effusions of patients with malignant and nonmalignant disease. *Cancer* **85**: 178–187

Millaure B, Shawver LK, Plate KH, Risau W and Ullrich A (1994) Glioblastoma growth inhibited in vivo by a dominant-negative Flk-1 mutant. *Nature* **367**: 576–579

Nakanishi Y, Kodama J, Yoshinouchi M, Tokumo K, Kamimura S, Okuda H and Kudo T (1997) The expression of vascular endothelial growth factor and transforming growth facotor-beta associates with angiogenesis in epithelial ovarian cancer. *Int J Gynecol Pathol* **16**: 256–262

Olson TA, Mohanaraj D, Carson LF and Ramakrishnan S (1994) Vascular permeability factor gene expression in normal and neoplastic human ovaries. *Cancer Res* **54**: 276–280

Orre M, Lotfi-Miri M, Mamers P and Rogers PAW (1998) Increased microvessel density in mucinous compared with malignant serous and benign tumours of the ovary. *Br J Cancer* **77**: 2204–2209

Orre M and Rogers P (1999) VEGF, VEDFR-1, VEGFR-2, mivcrovessel density and endothelial cell proliferation in tumours of the ovary. *Intl J Cancer* **84**: 101–108

Paley PJ, Staskus KA, Gebhard K, Mohanraj D, Twiggs LB and Carson LF et al (1997) Vascular endothelial growth factor expression in early stage ovarian carcinoma. *Cancer* **80**: 98–106

Peterson F (1988) Annual Report on Gynecological Cancer of FIGO, Vol. 20. Panorama Press: Stockholm

Senger DR, Galli SJ, Dvorak AM, Perruzzi CA, Harvey VS and Dvorak HS (1983) Tumor cells secrete a vascular permeability factor that promotes accumulation of ascites fluid. *Science* **219**: 983–985

Serov SF, Scully RE and Sobin LH (1973) Histological typing of ovarian tumours. International Histological Classification of Tumors, No 9. World Health Organization: Geneva

Sowter HM, Corps AN, Evans AL, Clark DE, Charnock-Jones DS, Smith SK (1997) Expression and localization of the vascular endothelial growth factor family in ovarian epithelial tumours. *Lab Invest* **77**: 607–614

 *© 2000 Cancer Research Campaign*

Tempfer C, Obermair A, Hefler L, Haeusler G, Gitsch G and Kainz C (1998) Vascular endothelial growth factor serum concentrations in ovarian cancer. *Obstet Gynecol* **92**: 360–363

Terman BI, Dougher-Vermazen M, Carrion ME, Dimitrov D, Armellino DC, Gospodarwics D and Bohlen P (1992) Identification of the KDR tyrosine kinase as a receptor for vascular endothelial cell growth factor. *Science* **255**: 989–991

Tischer E, Mitchell R, Hartman T, Silva M, Gospodarowicz D, Fiddes JC, Abraham JA (1991) The human gene for vascular endothelial growth factor. Multiple protein forms are encoded through alternative axon splicing. *J Biol Chem* **266**: 26031–26037

Weidner N, Semple JP, Welch WR and Folkman J (1991) Tumour angiogenesis and metastasis: correlation in invasive breast carcinoma. *N Engl J Med* **324**: 1–8

Weidner N and Folkman J (1996) Tumour vascularity as a prognostic factors in cancer. In: *Important Advances in Oncology*, VT De Vita VT, Hellman S and SA Rosenberg SA. (eds), pp. 167–190. Lippincott-Raven: Philadelphia

Yamamoto S, Konishi I, Mandai M, Kuroda H, Komatsu T and Nanbu K, et al. (1997) Expression of vascular growth factor (VEGF) in epithelial ovarian neoplasms: Correlation with clinicopathology and patient survival, and analysis of serum VEGF levels. *Br J Cancer* **76**: 1221–1227

*© 2000 Cancer Research Campaign*
